# Arsenic leads to autophagy of keratinocytes by increasing aquaporin 3 expression

**DOI:** 10.1038/s41598-021-96822-6

**Published:** 2021-09-01

**Authors:** Sebastian Yu, Ling-Hau Li, Chih-Hung Lee, Palaniraja Jeyakannu, Jeh-Jeng Wang, Chien-Hui Hong

**Affiliations:** 1 Graduate Institute of Clinical Medicine, College of Medicine, Kaohsiung Medical University, Kaohsiung, Taiwan; 2 Department of Dermatology, Kaohsiung Medical University Hospital, Kaohsiung Medical University, Kaohsiung, Taiwan; 3 Department of Dermatology, College of Medicine, Kaohsiung Medical University, Kaohsiung, Taiwan; 4 Department of Dermatology, Kaohsiung Chang Gung Memorial Hospital and Chang Gung University College of Medicine, Kaohsiung, Taiwan; 5 Department of Medicinal and Applied Chemistry, Kaohsiung Medical University, Kaohsiung, Taiwan; 6 Department of Dermatology, Kaohsiung Veterans General Hospital, Kaohsiung, Taiwan; 7 Department of Dermatology, National Yang-Ming University, Taipei, Taiwan; 8 Department of Dermatology, School of Medicine, National Yang Ming Chiao Tung University, Taipei, Taiwan

**Keywords:** Cancer, Environmental sciences, Molecular medicine

## Abstract

Exposure to arsenic, a ubiquitous metalloid on Earth, results in human cancers. Skin cancer is the most common arsenical cancers. Both autophagy and aquaporin pathway are known to promote carcinogenesis. However, the mechanisms by which arsenic regulates aquaporin and autophagy in arsenical skin cancers remain elusive. This study aims to address how arsenic regulates aquaporin-3, the predominant aquaporin in epidermal keratinocytes, and how this process would induce autophagy. Quantitative real-time PCR and immunofluorescence were used to measure the expression of aquaporin 3 in arsenical skin cancers and arsenic-treated keratinocytes. Beclin-1 expression and autophagy were measured. We examined if blocking aquaporin 3 could interfere arsenic-induced autophagy in keratinocytes. Expression of aquaporin 3 is increased in arsenical cancers and in arsenic-treated keratinocytes. Arsenic induced autophagy in primary human keratinocytes. Notably, the arsenic-induced autophagy was inhibited by pretreatment of keratinocytes with aquaporin inhibitors Auphen or AgNO_3_, or RNA interference against aquaporin 3. The data indicates that the aquaporin 3 is an important cell membrane channel to mediate arsenic uptake and contributes to the arsenic-induced autophagy.

## Introduction

Arsenic is a ubiquitous metalloid on Earth’s crust^1^. Chronic arsenic exposure remains a global health issue^2^. Arsenic exposure in humans can lead to various cancers, including liver, lung, kidney, bladder, and skin cancers^3^. Skin cancer is the most common arsenic-induced cancer^4^. Among skin cancerous changes, Bowen’s disease (BD) is the most common and the earliest cancerous skin lesion derived from epidermal keratinocytes^4^. Therefore, research for arsenic-induced Bowen’s disease provides a platform to investigate arsenic-induced early carcinogenesis.

Arsenic-induced BD (As-BD) can develop into invasive cancers. The characteristic histological features of arsenic-induced BD include full-layer epidermal dysplasia and individual cell apoptosis^5^. The full-layer epidermal dysplasia results from aberrant keratinocyte differentiation. Keratinocyte differentiation is delicately regulated by calcium homeostasis^6^. More specifically, the calcium-sensing receptor (CaSR) as G protein-coupled receptor (GPCR) senses extracellular calcium changes and activates a plethora of calcium channels such as transient receptor potential (TRP) channels and Orai1. Dysregulation of intracellular calcium concentration in keratinocytes leads to aberrant keratinocyte differentiation. Our previous study shows arsenic impairs calcium propagation in keratinocyte that can be partially restored by reducing agents such as hydrogen-enriched water^7^. Dysregulation of calcium homeostasis that leads to abnormal keratinocyte differentiation is one of the mechanisms underlying arsenic-induced carcinogenesis.

Arsenic not only induces apoptosis of keratinocytes but have effects on monocyte-macrophage system and lymphocytes^2,8^. Briefly, we previously showed that bone marrow-derived dendritic cell (DC) from As-BD patients have impaired CCL21-mediated DC migration. The STAT3-VEGF axis in keratinocytes inhibits DC migration in the microenvironment of As-BD and thus explains why the DC migration is impaired in arsenical cancers^9^.

Another mechanism underlying As-BD is mitochondrial biogenesis^10,11^. In As-BD, mitochondrial biogenesis-related genes are overexpressed. Enhanced mitochondrial biogenesis leads to aberrant keratinocyte proliferation, and blocking of mitochondrial function abrogates arsenic-induced keratinocyte proliferation^10^. Epigenetic modification also plays a role in arsenic-induced carcinogenesis^12^. In an skin-equivalent organotypic culture model consisting of keratinocytes, fibroblasts, and peripheral blood mononuclear cells, arsenic treatment leads to pathognomonic characteristics of As-BD. By using this model, we identified arsenic induces epigenetic modification of E2F1 promotor, which leads to centromere amplification and subsequent caspase-8-mediated apoptosis of keratinocytes^12^.

An important pathognomic feature of As-BD is the coexistence of uncontrolled cell proliferation, abnormal differentiation, along with individual cell death among the epidermal keratinocytes. Autophagy is reported to be involved in carcinogenesis of skin. A report showed autophagy gene ATG7 regulates ultraviolet radiation-induced inflammation and skin tumorigenesis^13^. For biological samples, beclin-1 protein is a well-established biomarker to measure the development of autophagy^14^. In addition to beclin-1 expression, the autophagic flux is used to detect autophagy in various diseases^15^. LC3 proteins are cleaved by Atg4 to become LC3-I, which then conjugates to phosphatidylethanolamine to form LC3-II during autophagy^15^. Thus, LC3 decreases while LC3-II increases in autophagy induction^16^. Hence, the ratio of LC3-II/LC3-I could be another useful marker for autophagic flux. On the other hand, p62 is a link between LC3 and ubiquitinated substrates and is degraded during autophagy. Therefore, autophagic activation correlates with a decreased p62 level, while autophagic suppression correlates with an increased p62 level^17^. While autophagy is regarded as an important tumor facilitator, the exact role of autophagy in As-BD remains to be elucidated. Another mechanism that may contribute to skin carcinogenesis is transportation of arsenic. Transport systems responsible for uptake and toxicity of inorganic arsenic can be divided into trivalent arsenite uptake and pentavalent arsenate uptake^18^. Because arsenic is not required for life, there seem no arsenic-specific uptake systems. Pentavalent arsenate is taken into most cells adventitiously by phosphate uptake systems while trivalent arsenite is taken into most cells primarily via aquaglyceroporins or sugar permeases. Trivalent arsenites are more toxic than the pentavalent arsentes. The first trivalent arsenite uptake system was identified in 1997^19^. As(III) and trivalent antimony, Sb(III), were demonstrated to be transported into cells of Escherichia coli by the glycerol channel GlpF, which belongs to the aquaporin superfamily^19,20^.

Since mechanisms contributing to As-BD such as epigenetic regulation^21^ and mitochondria^22^ are also related to aquaporin 3 (AQP3), it is intriguing to explore if AQP3 plays a role in the pathogenesis of As-BD. More specifically, we aim at investigating if arsenic is transported into keratinocytes through AQP3 in As-BD and, if so, how this process would induce autophagy to facilitate carcinogenesis. Herein, we showed expression of aquaporin 3, the most common aquaporin in keratinocytes, is up-regulated in arsenical skin cancers and arsenic-treated keratinocytes. Arsenic induces autophagy in keratinocytes, and blocking AQP3 interferes arsenic-induced autophagy. These data indicate that AQP3 is an important membrane protein to mediate arsenic uptake in keratinocytes and that AQP3 contributes to the arsenic-induced autophagy.

## Results

### Increased AQP3 expression in As-BD

**Figure 1:**
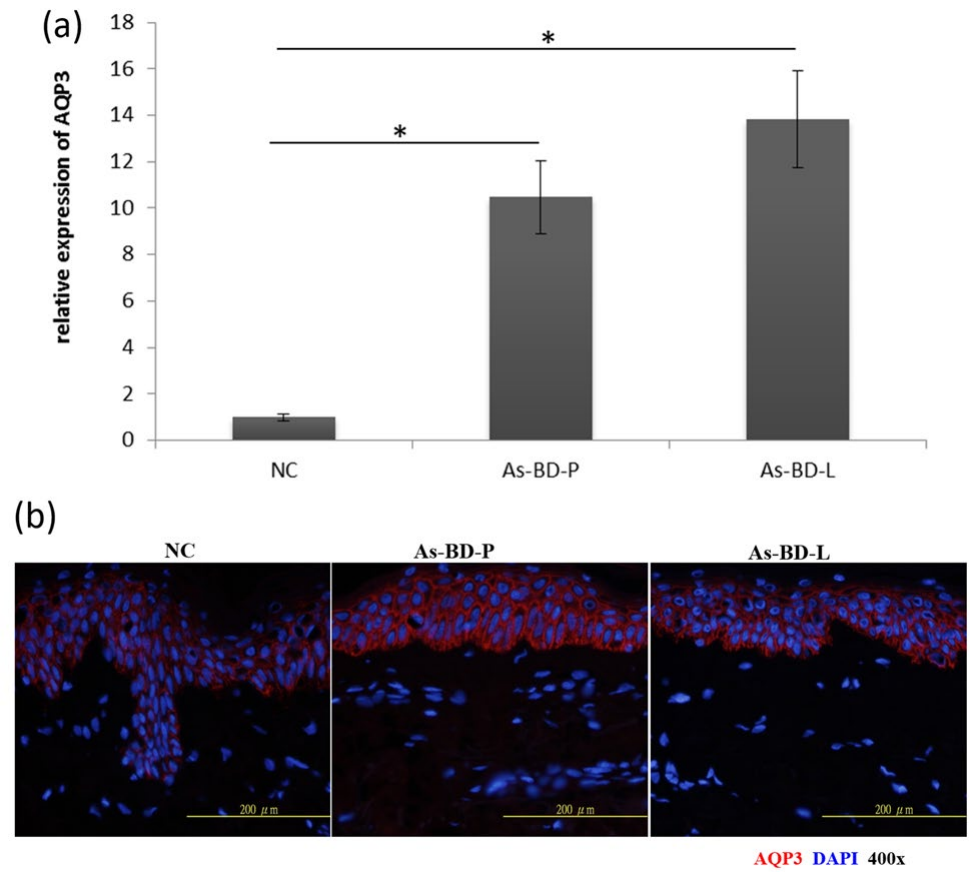
Expression of aquaporin 3 (AQP3) is increased in arsenical Bowen’s disease(As-BD). (**a**) Quantitative real-time PCR showed that mRNA expression level of AQP3 significantly increased in lesional and perilesional skin as compared with normal control skin. N = 15 and 10 for As-BD and NC (normal controls), respectively. *P < 0.05. (**b**) Representative immunofluorescence showed positive AQP3 expression in lesional and perilesional skin of arsenical Bowen’s disease while AQP3 expression was subtle in normal control. *BD* Bowens’ disease. *P* perilesional skin. *L* lesional skin. *NC* normal control.

The human As-BD tissues showed typical full-layer epidermal dysplasia, epidermal hyperplasia, and individual cell apoptosis. Because AQP3 may facilitate arsenic absorption into keratinocytes, we asked whether AQP3 was increased in As-BD skin lesions (Fig. 1). The result of quantitative real-time PCR showed that mRNA of *AQP3* is increased in lesional and perilesional As-BD than that in normal control skin (Fig. 1a). We further examined if increased AQP3 expression could be reproduced in protein level. By using immunofluorescence, the results showed that samples harvested from lesional and perilesional As-BD showed positive immunofluorescence of AQP3 (Fig. 1b). These experiments confirmed that expression of AQP3 is increased in As-BD.

### Increased AQP3 expression in arsenic-treated keratinocytes

**Figure 2:**
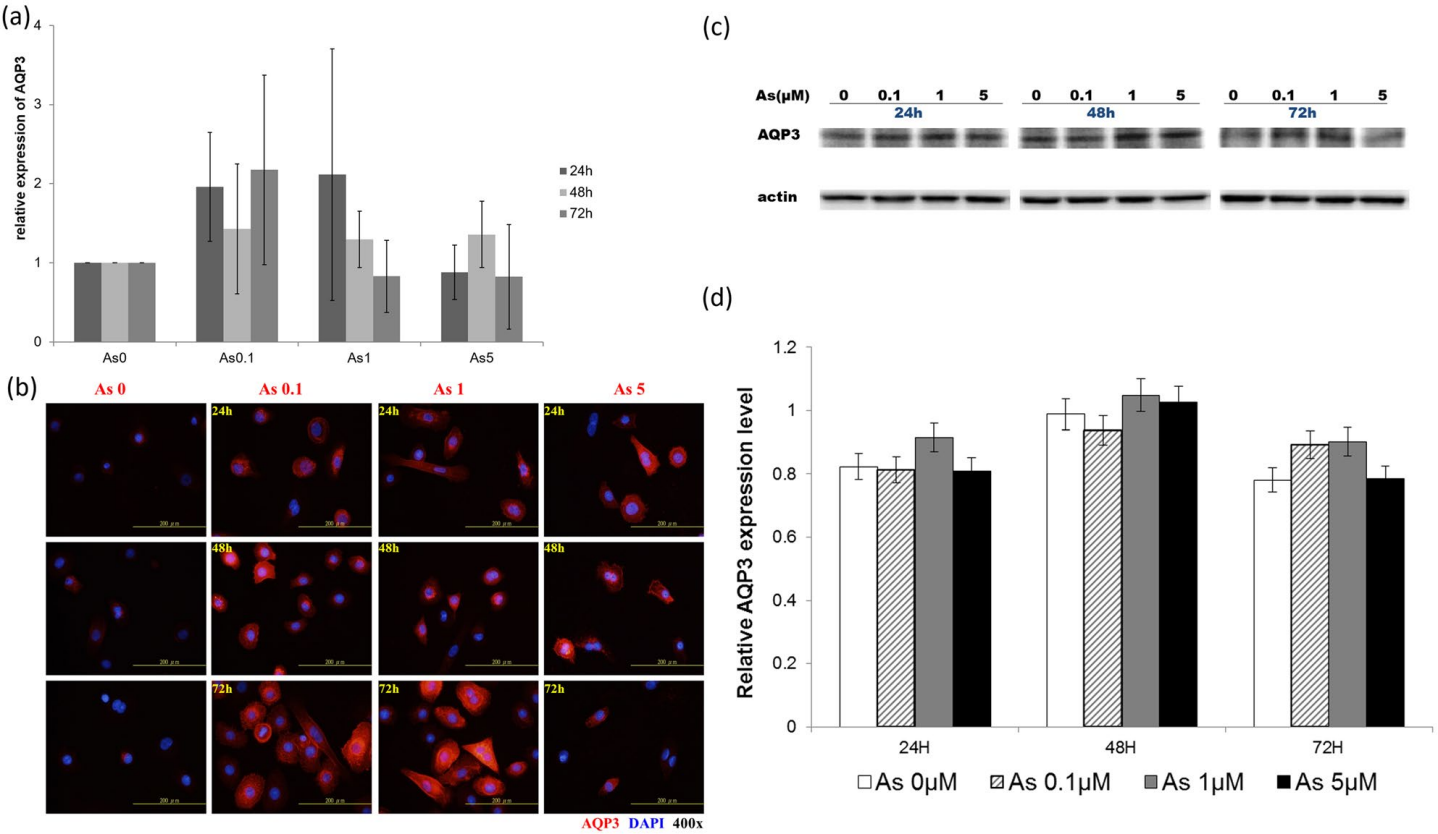
Increased expression of aquaporin-3 (AQP3) in arsenic-treated keratinocytes. (**a**) Quantitative real-time PCR showed a partial increase in mRNA of AQP3 in arsenic-treated primary keratinocytes although the difference in mRNA expression among different concentrations and time points didn’t reach statistical significances. One of the representative experiments with triplicates was shown. (**b**) By immunofluorescence exam, arsenic-treated primary keratinocytes had increased protein expression of AQP3 at arsenic concentrations of 0.1 and 1 µM after treatment for 24 h, 48 h, 72 h, while various intensity of immunofluorescence of AQP3 was observed at arsenic concentrations of 5 µM, suggesting a partial toxicity of arsenic at 5 µM after 48 and 72 h treatment. Representative pictures are shown. (c) Western blotting showed total protein expression of AQP3 in arsenic-treated primary keratinocytes when compared with those without arsenic treatment. Two repeated experiments were performed, and a representative blot is shown. (**d**) Densitometry of AQP3 expression from (**c**).

To further investigate the mechanisms how expression of AQP3 may contribute to pathogenesis of As-BD, we used arsenic-treated keratinocytes to measure the mRNA and protein expression of AQP3 in *vitro* (Fig. 2). Consistent with what was observed in As-BD, real-time PCR analysis showed slight upregulated *AQP3* in keratinocytes treated with arsenic at 0.1 µM or 1 µM. The *AQP3* upregulation was less prominent in keratinocytes pretreated with 5 µM (Fig. 2a). Interestingly, the protein expression of AQP3 was increased in keratinocytes treated with arsenic at 0.1 µM and 1 µM at 24, 48, and 72 h as shown by immunofluorescent exam. For keratinocytes treated with arsenic at 5 µM, immunofluorescence exam showed an increased AQP3 expression at 24 h while the intensity of immunofluorescence was lesser at 48 and 72 h, suggesting a partial toxicity of arsenic or other compensatory mechanisms after 48 h treatment at this concentration (Fig. 2b). While immunofluorescence (Fig. 2b) and quantitative real-time PCR (Fig. 2a) both showed increased AQP3 expression, western blotting (Fig. 2c,d; Supplementary Fig. S1-S4) from the whole cell lysate did not show significant difference of AQP3 expression. This discrepancy may result from the increased AQP3 expressions mainly on cell membrane that were detected better by immunofluorescence than by western blot. These data suggested that arsenic may induce expression of AQP3 on keratinocyte surfaces while its biological effect remains to be clarified.

### *AQP3* siRNA transfection partially inhibited arsenic-induced expression of AQP3 and autophagy

**Figure 3:**
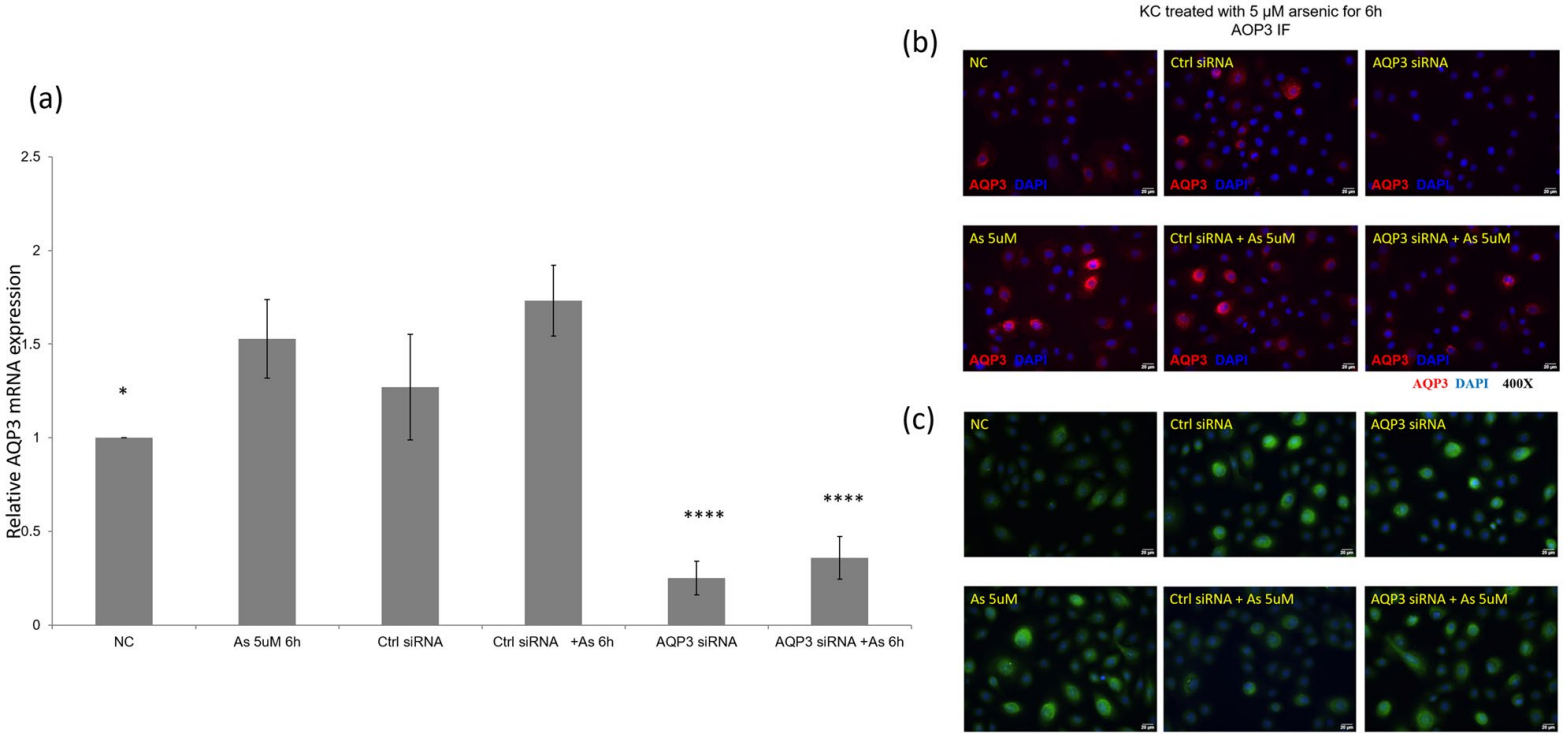
Arsenic-induced autophagy was in part alleviated by RNA interference targeting aquaporin 3 (AQP3). (**a**) Compared with control siRNA, AQP3 siRNA significantly reduced AQP3 mRNA expression in keratinocytes treated with arsenic at a concentration of 5 µM for 6 h. Signal of AQP3 expression was measured in duplicate by quantitative real-time PCR. *P < 0.05. ****P < 0.0001, compared with the arsenic 5 µM 6 h group. (**b**) Consistent with quantitative real-time PCR findings, AQP3 siRNA reduces immunofluorescence of AQP3 in arsenic-treated keratinocytes. (**c**) Keratinocytes treated with arsenic shows arsenic-induced autophagy, as detected by an autophagy kit (ab139484, Abcam). AQP3 siRNA partially reduced arsenic-induced autophagy.

We then asked whether AQP3 mediated autophagy induced by arsenic. To do so, we transfected cells with *AQP3* siRNA and see whether blocking AQP3 would inhibit arsenic-induced autophagy. To avoid off-target effects due to long incubation and transfection process, we examined results at 6 h with higher arsenic concentration (5 µM). As expected, transfection of *AQP3* siRNA significantly reduced *AQP3* mRNA expression in keratinocytes treated with arsenic at 5 µM for 6 h as compared to control siRNA (Fig. 3a) (*P* < 0.0001). Immunofluorescence exam showed arsenic induced the expression of AQP3 in a small fraction of cells, while *AQP3* siRNA transfection inhibited the surface expression of AQP3 in arsenic-treated keratinocytes (Fig. 3b). Autophagy assay kit also showed that arsenic induced autophagy in keratinocytes. Reduced autophagy was observed in keratinocytes transfected with RNA interference against *AQP3* (Fig. 3c). These data indicated that arsenic-induced autophagy is mediated, at least partially, by AQP3.

### Arsenic-induced autophagy was inhibited by pretreatment with AgNO_3_, a chemical inhibitor for AQP3

**Figure 4:**
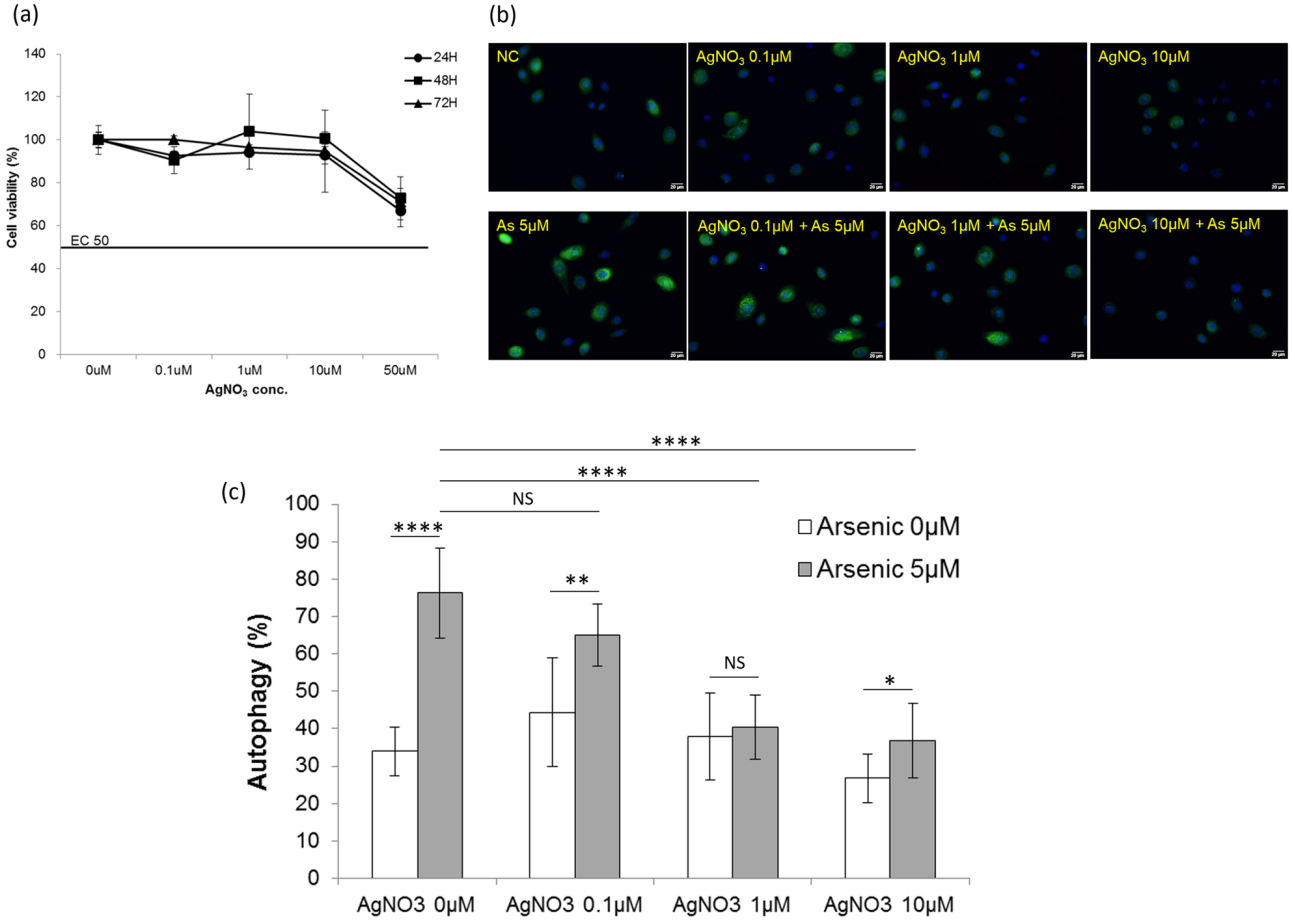
Pretreatment with AgNO_3_, an aquaporin 3 (AQP3) inhibitor, suppresses arsenic-induced autophagy. (**a**) The EC50 of which AgNO_3_ inhibits AQP3 activity is > 50 µM. (**b**) Keratinocytes were pretreated with AgNO_3_ at a concentration of 0.1, 1, or 10 µM for 15 min. The autophagy was measured by an autophagy assay kit (ab139484, Abcam). Compared with those without pretreatment of AgNO_3_, keratinocytes with AgNO_3_ pretreatment had reduced arsenic-induced autophagy. (**c**) Without pretreatment of AgNO_3_, arsenic at a concentration of 5 µM induced autophagy. After pretreatment of AgNO_3_, the percentages of keratinocytes with autophagy decreased. The inhibition effect of AgNO_3_ on arsenic-induced autophagy showed dose-dependent effect. NS, no statistical significance. *P < 0.05. **P < 0.01. ****P < 0.0001.

The cell toxicity of AgNO_3_ is low below 50 µM (Fig. 4a). To compile further evidences whether AQP3 might mediate arsenic-induced autophagy in keratinocytes, we pretreated cells with AgNO_3_, a chemical inhibitor for AQP3, and investigated whether arsenic-induced autophagy could be inhibited. The autophagy was measured by an autophagy assay kit, showing arsenic at 5 µM significantly induced autophagy (Fig. 4b). Notably, after pretreatment with AgNO_3_, the percentages of autophagic keratinocytes were decreased significantly, particularly in those pretreated with AgNO_3_ at 1 and 10 µM (Fig. 4b). Quantized data from the autophagy assay kit showed the inhibition effect of AgNO_3_ on arsenic-induced autophagy was dose-dependent (Fig. 4c).

### Arsenic-induced *AQP3* expression was reduced by pretreatment of Auphen, another chemical inhibitor for AQP3

**Figure 5:**
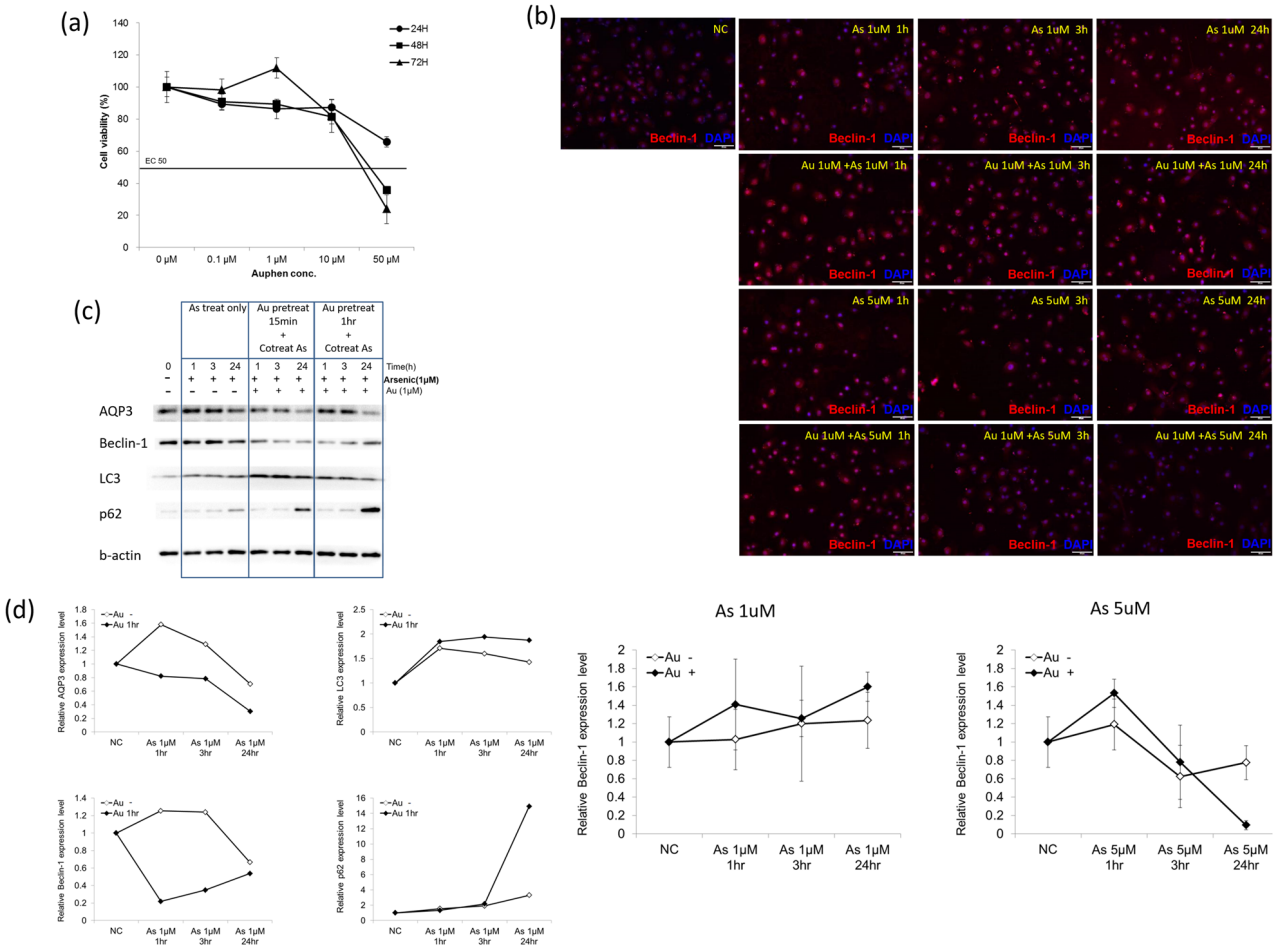
Auphen inhibits arsenic-induced AQP3 and beclin-1 expression. (**a**) The cell viability of Auphen is low at concentrations below 10 µM (EC50 > 10 µM). (**b**) Keratinocytes were pretreated with or without Auphen (1 µM) for 1 h, and then co-treated with arsenic (1 or 5 µM) for 1, 3, and 24 h. Immunofluorescent exam was used to measure beclin-1 expression. In cells treated with arsenic at 1 µM, the beclin-1 expression was not significantly different whether cells were pretreated with Auphen. In cells treated with arsenic at 5 µM, Auphen pretreatment reduced the expression of beclin-1 at 24 h after arsenic treatment. (**c**) Keratinocytes were pretreated with Auphen (1 µM) for 15 min or 1 h and then co-treated with arsenic (1 µM) for 1, 3, and 24 h. The protein expressions of AQP3, beclin-1, LC3, and p62 were measured by Western blot. Two repeated experiments were performed, and a representative blot is shown. NC: normal control. (**d**) Densitometry of expressions of AQP3, beclin-1, LC3, and p62 from (**c**). Since the trend from Auphen pretreatment for 15 min and 1 h appeared similar, only 1 h Auphen pretreatment data is shown.

Since AgNO_3_ might have off-targeted effects other than AQP3 inhibition, we synthesized Auphen, another chemical inhibitor, that was known to inhibit AQP3^23^, and tested whether this compound could inhibit autophagy induced by arsenic. Our result showed that cell viability of Auphen was low (Fig. 5a), with a concentration leading to 50% cell viability (EC50) lies between 10 and 50 µM. In cells treated with arsenic at 1 µM, the beclin-1 expression as measured by immunofluorescence was not significantly different whether cells were pretreated with Auphen or not. In cells treated with arsenic at 5 µM, Auphen pretreatment for 1 h reduced the expression of beclin-1 at 24 h after arsenic treatment (Fig. 5b). We next determined whether arsenic-induced autophagy could be rescued by Auphen. To do that, we measured the expression of autophagy-related markers, including beclin-1, LC3, and p62 by western blot. Consistent with immunofluorescence findings, western blot showed that enhanced expression of beclin-1 as well as AQP3 by arsenic was inhibited by pretreatment of Auphen and the inhibition occurred as early as 1 h after arsenic treatment (Fig. 5c,d; Supplementary Fig. S5-S9). LC3 was induced by arsenic, however, no significant decreases were found in cells pretreated with Auphen. Intriguingly, p62 expression was not changed significantly in arsenic-treated keratinocytes up to 3 h and its expression was even robustly increased in Auphen-pretreated keratinocytes 24 h after arsenic treatment (Fig. 5d).

## Discussion

AQP3 plays a role in hydration in epidermis, and the expression of AQP3 is changed in skin diseases such as atopic dermatitis and psoriasis^24,25^. In the present study, we show AQP3 expression is increased in As-BD skin lesions and arsenic-treated keratinocytes. While arsenic induces autophagy in keratinocytes, this can be reserved by RNA interference against AQP3 as well as chemical inhibitors for AQP3, including AgNO_3_ and Auphen.

Considering that arsenic exposure due to drinking water contamination remains to be a global issue not only in undeveloped countries but also in developed countries such as USA^2^, any interventions that can reduced uptake of arsenic into keratinocytes provides potential therapeutics to prevent As-BD and possibly arsenic-related cancers.

In views of that drinking water with potential arsenic contamination cannot be rapidly changed due to various issues such as government infrastructural construction and unclear and/or undisclosed quantification of arsenic concentration of drinking water in various areas of the world, the development of a compound or medication that can prevent uptake of arsenic into cells is a practical and useful strategy to reduce arsenic-related cancers. In the present study, we identify that Auphen, also known as 1,10-phenanthroline gold(iii) dichloride, inhibits arsenic-induced autophagy. This compound or other chemicals may be used as a medication to prevent reuptake of arsenic into human cells and subsequent arsenical cancers, although further preclinical studies need to be done to further support this prevention strategy, including short term toxicity, long term safety, and animal experiments.

Our study showed that both the chemical inhibitors for aquaporin, AgNO_3_ and Auphen, could inhibit the autophagy or beclin-1 expression in arsenic-treated keratinocytes. However, in Auphen pretreatment experiments (Fig. 5), although LC3 was induced by arsenic, this induction was not decreased in cells pretreated with Auphen. More intriguingly, p62 expression was dramatically increased in Auphen-pretreated keratinocytes 24 h after arsenic treatment. It is possible that measurement of p62 expression and LC3 by western blot may not be the optimal way to measure autophagy. The autophagic flux is at best confirmed by GFP-mRFP-LC3, not by western-blotting of LC3 and p62. In addition to its role in autophagy, p62 may mediate keratinocyte proliferation in psoriasis, a disease with hyperproliferative epidermis by keratinocytes^26^. P62 is involved in the arsenic-induced keratinocyte proliferation too^27^. Therefore, the induction of p62 expression in Auphen-pretreated keratinocytes at 24 h after arsenic treatment may have biological roles other than autophagy induced by arsenic. Auphen may exert biological effects other than inhibition of AQP3. The bottom line is that we showed Auphen, a chemical inhibitor for AQP3, inhibited arsenic-induced expression of beclin-1 and autophagy.

There are limitations of the present study. In this study, we didn’t measure arsenic concentrations of tissue samples from the patients with As-BD. Although, technically, arsenic concentrations can be measured from human samples such as hairs and nails, the linear correlations between tissue concentrations and skin lesions are not high^28^. In addition, arsenic concentrations in well water vary, and it is difficult to quantify arsenic exposure in each patient. Therefore, we use primary keratinocytes to perform experiments to quantify arsenic exposure and examine autophagy markers to clarify the effects of arsenic on keratinocytes. Finally, as mentioned previously, the autophagic flux is at best confirmed by GFP-mRFP-LC3, not by western-blotting of LC3 and p62.

In conclusions, we demonstrate that a new mechanism how AQP3 facilitates arsenic uptake and may lead to As-BD and that this mechanism can be inhibited by an Au compound. The present study highlights a potential therapeutic for arsenical cancers. More pre-clinical and human clinical studies are warranted to validate the role of Au compound to reduce incidence of As-BD and other arsenical cancers.

## Methods

### Study subjects

Twenty-five biopsy-proven As-BD tissue samples were obtained from 15 patients (10 males and 5 females, age range: 62–82 years). The As-BD was diagnosed based on the typical pathological findings from human subjects, which showed full-layer epidermal dysplasia, epidermal proliferation, and individual cell apoptosis. All patients have arsenic well water exposure history since childhood. Because vitiligo is a confounder of AQP3 expression, and therefore patients with vitiligo were excluded in our present study^29^. Control skin of As-BD patients was harvested from patients’ normal skin by minimal skin biopsy (for routine skin surgery to remove benign skin tumors). The procedures were described in the IRB, and all patients signed informed consent. Normal human primary keratinocytes were obtained from adult foreskin through routine circumcision. Written informed consent was obtained from all participants to obtain As-BD tissues and primary keratinocytes. This work has received approval for research ethics from Institutional Review Board at Chang Gung Memorial Hospital (104-9380A3) and a proof/certificate of approval is available upon request. All methods were performed in accordance with the relevant guidelines and regulations.

### Primary keratinocyte culture

The method for keratinocyte cultivation is described as previously reported^7^. Briefly, skin specimens were washed with phosphate-based saline (PBS), cut into small pieces, and incubated in medium containing 0.25% trypsin (Gibco, Grand Island, NY) overnight at 4℃. The epidermal sheet was lifted from the dermis using fine forceps. The epidermal cells were centrifuged (500 g, 10 min) and the pellets dispersed into individual cells by repeated aspiration. The keratinocytes were gently resuspended in keratinocyte-SFM (serum-free medium) (Gibco) containing 25 lg/ml bovine pituitary extract (BPE) and 5 ng/ml recombinant human epidermal growth factor (rhEGF). The medium was changed every two days. Keratinocytes at the third passage were then grown in keratinocyte-SFM medium free of supplements 24 h before the experiments.

### Quantitative real-time PCR

Quantification and purification of the RNA were as previously reported^30^. Briefly, the RNA was measured by A260/A280 absorption (NanoDrop spectrophotometer; Thermo Fisher Scientific, Waltham, MA, USA). RNA samples with ratios greater than 1.7 were stored at − 70 °C for further analysis. Extracted RNA (1 μl) was then subjected to PCR amplification using MPCR kits (Maxim Biotech, San Francisco, CA, USA) according to the manufacturer’s instructions^30^. Quantitative real-time PCR was performed using LightCycler 96 real-time PCR system (Roche, Mannheim, Germany). The *AQP3* and reference gene β-actin primers were obtained from Genomics (New Taipei City, Taiwan;β-actin -F: GGCGGCACCACCATGTACCCT, β-actin -R: 5’-AGGGGCCGGACTCGTCATACT-3’, hAQP3-F: GCAGCCTGTCCATCTGTG, hAQP3-R: ACCCTACTTCCCAAAAGCC).

### Immunofluorescence of AQP3 and beclin-1

The AQP3 and beclin-1 expression were measured by adding the primary antibodies (AQP3: ab125219, Abcam, Cambridge, UK; beclin-1: GTX31722, GeneTex, Irvine, CA, USA), followed by the secondary antibody of goat Alexa Fluor 568-conjugated anti-mouse IgG (H + L) (A11004, Invitrogen, Waltham, MA, USA). Beclin-1 expression reflects autophagy. Nuclei were stained with DAPI (Fluoromount-G; Sigma, St. Louis, MO, USA). Fluorescence images were acquired by Olympus BX53 microscope and analyzed using ZEN software (Zeiss, Oberkochen, Germany)^31^.

### Autophagy detection

The autophagy was detected by an autophagy assay kit (ab139484, Abcam). The percentage of autophagy cells was averaged from the number of autophagy kit-stained cells divided by the number of DAPI-stained cells from 4 high power fields.

### Western blotting

The proteins were then extracted based on the manufacturer's instructions. Equivalent amounts of protein per sample were electrophoretically resolved on 10% polyacrylamide gels and transferred onto 0.2 mm nitrocellulose membranes. Proteins were probed overnight with primary antibodies against AQP3 (1:1000; ab125219, Abcam), beclin-1 (1:1000; GTX 31,722, GeneTex), LC3B (1:1000; CST 3868, Cell Signaling Technology, Danvers, MA, USA), actin (1:5000, MAB1501, Millipore, Burlington, MA, USA). The nitrocellulose membranes were then incubated with an appropriate horseradish peroxidase-conjugated secondary antibody for 1 h at room temperature, and the immunoreactivity was observed by enhanced chemiluminescence detection, a semiquantitative assay through blot dosimetry. Anti-β-actin (Epitomics, Cambridge, MA, USA) was used to check for equal loading of protein between wells^25^.

### Cell viability

To determine the cytotoxicity of AgNO_3_ and Auphen, we treated keratinocytes with different concentrations of both compounds. We then measured the cell viability by Cell Counting Kit-8 (CCK-8, Sigma-Aldrich, St Louis, MO, USA) to estimate EC50.

### Inhibition of *AQP3* by small interfering RNA (siRNA)

The siRNA protocol was adapted from the previously reported^32^. Briefly, cells were seeded to be 70–90% confluent before transfection. Cells were transfected with *AQP3* siRNA or negative control (NC) siRNA (both from Dharmacon, Lafayette, CO, USA) using Lipofectamine 3000 Transfection Reagent according to instructions from the manufacturer (Thermo Fisher Scientific). Lipofectamine 3000 Reagent was diluted in siRNA-containing serum-free medium for 15 min at room temperature. The siRNA–lipid complex was then added to the cells for 6 h. Thereafter, the siRNA-lipid complex was replaced and incubated with FBS at 37 °C in a humid atmosphere with 5% CO2 before the subsequent western blot and quantitative PCR.

### Inhibition by AgNO_3_

Keratinocytes were treated with AgNO_3_, an aquaporin 3 (AQP3) inhibitor, to examine if AgNO_3_ suppresses arsenic-induced autophagy. Keratinocytes were pretreated with AgNO_3_ at a concentration of 0.1, 1, or 10 µM for 15 min. Keratinocytes were divided into 2 groups based on pretreatment or without pretreatment of AgNO_3_. Arsenic at a concentration of 5 µM or 0 µM (control group) was used to treated keratinocytes with or without AgNO_3_ pretreatment to examine the effect of AgNO_3_ on arsenic-induced autophagy.

### Inhibition by Auphen (C_12_H_8_AuCl_2_N_2_)

Next, we used Auphen, an Au chemical compound that inhibits arsenic-induced *AQP3* expression, to examine if chemical inhibition has similar results with siRNA inhibition and AgNO_3_ inhibition. Auphen is synthesized accordingly^33,34^. Among gold-based metal compounds, Auphen has the most active binding activity on AQP3^23^. While other AQP3 inhibitors may be toxic, Auphen shows a non-toxic inhibitory effect and therefore has more potential for clinical use^23^. Auphen used in this experiments was synthesized by PJ and JJW with purity more than 99%.

### Statistical analysis

All data are expressed as mean ± standard deviation (SD). For multiple-group (more than two groups) comparisons, one-way analysis of variance (ANOVA) with Tukey post hoc test was used for multiple comparisons. A *P*-value less than 0.05 was considered statistically significant.

## Supplementary Information

Supplementary Information.
