# Supplementary material for: Arsenic leads to autophagy of keratinocytes by increasing aquaporin 3 expression
Source: Sci Rep. 2021 Sep 1;11:17523. doi: 10.1038/s41598-021-96822-6 (PMC8410848; doi:10.1038/s41598-021-96822-6)
Supplement: Supplementary file 1 — Supplementary Information. [file 41598_2021_96822_MOESM1_ESM.pdf]

Arsenic leads to autophagy of keratinocytes by increasing aquaporin 3 expression

Sebastian Yu[1,2,3], Ling-Hau Li[4], Chih-Hung Lee[4], Palaniraja Jeyakannu[5], Jeh-Jeng Wang[5], Chien-Hui Hong [6,7,8*]

[1]Graduate Institute of Clinical Medicine, College of Medicine, Kaohsiung Medical University, Kaohsiung, Taiwan

[2]Department of Dermatology, Kaohsiung Medical University Hospital, Kaohsiung Medical University, Kaohsiung, Taiwan

[3]Department of Dermatology, College of Medicine, Kaohsiung Medical University, Kaohsiung, Taiwan

[4]Department of Dermatology, Kaohsiung Chang Gung Memorial Hospital and Chang Gung University College of Medicine, Kaohsiung, Taiwan

[5]Department of Medicinal and Applied Chemistry, Kaohsiung Medical University, Kaohsiung, Taiwan

[6]Department of Dermatology, Kaohsiung Veterans General Hospital, Kaohsiung, Taiwan

[7]Department of Dermatology, National Yang-Ming University, Taipei, Taiwan

[8]Department of Dermatology, School of Medicine, National Yang Ming Chiao Tung University, Taipei, Taiwan

*Corresponding author:

Chien-Hui Hong, MD, PhD

Department of Dermatology, Kaohsiung Veterans General Hospital, Kaohsiung, Taiwan &

Associate Professor, Department of Dermatology, Faculty of Medicine, National Yang-Ming University, Taipei, Taiwan &

Associate Professor, Department of Dermatology, School of Medicine, National Yang Ming Chiao Tung University, Taipei, Taiwan

Tel: 886-7-3422121 ext 4300

E-mail: zieben@gmail.com

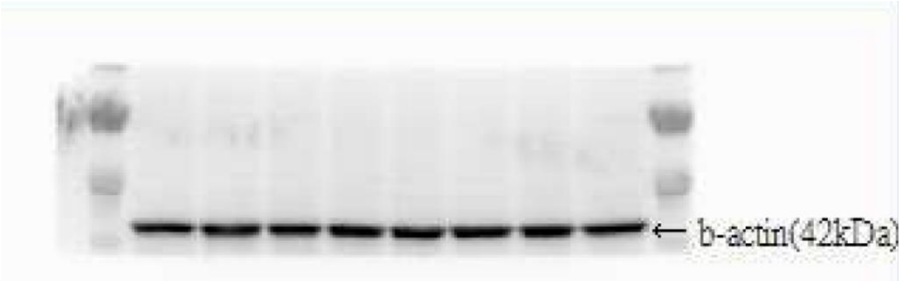

Supplementary Figure S1. Western blotting of b-actin of Figure 2c (24h, 48h).

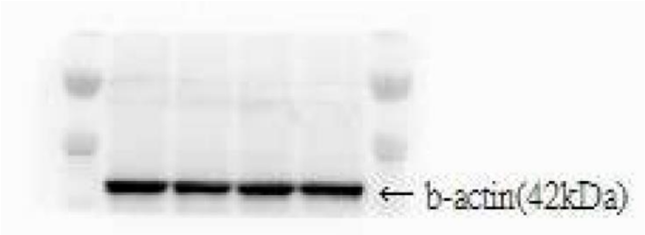

Supplementary Figure S2. Western blotting of b-actin of Figure 2c (72h).

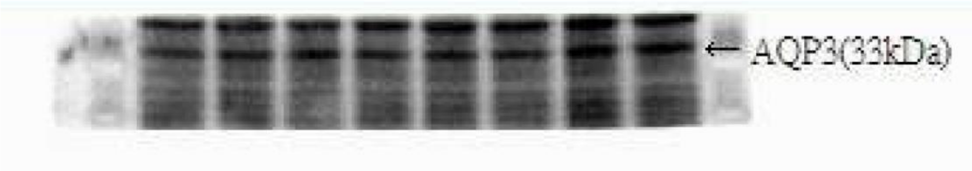

Supplementary Figure S3. Western blotting of AQP3 of Figure 2c (24h, 48h).

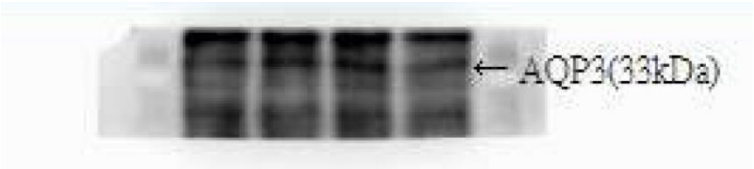

Supplementary Figure S4. Western blotting of AQP3 of Figure 2c (72h).

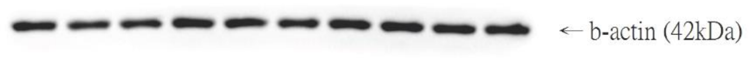

Supplementary Figure S5. Western blotting of b-actin of Figure 5c.

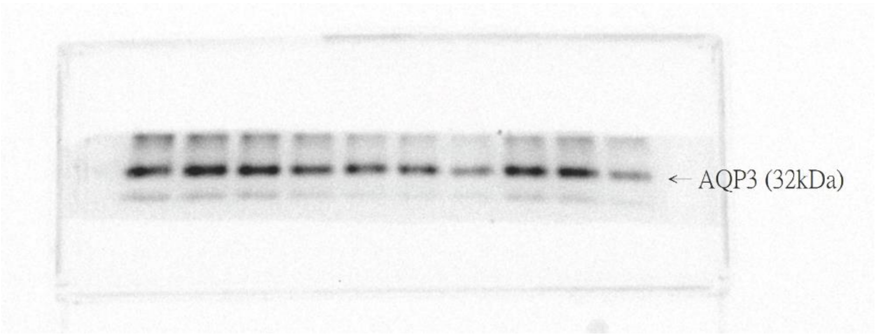

Supplementary Figure S6. Western blotting of AQP3 of Figure 5c.

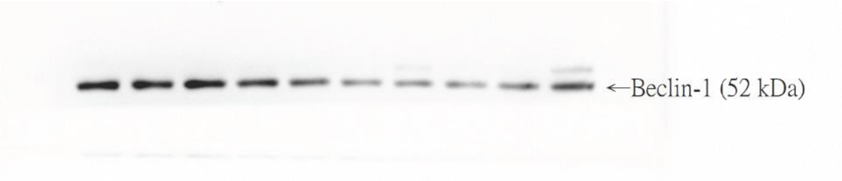

Supplementary Figure S7. Western blotting of beclin-1 of Figure 5c.

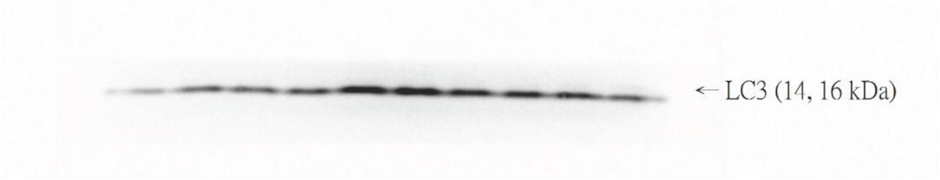

Supplementary Figure S8. Western blotting of LC3 of Figure 5c.

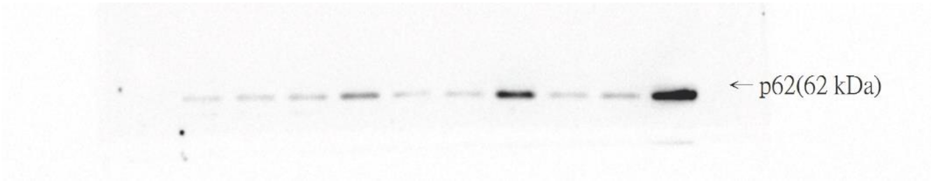

Supplementary Figure S9. Western blotting of p62 of Figure 5c.
